# Soluble B-Cell Maturation Antigen as a Prognostic Marker for Progression-Free Survival in Multiple Myeloma Treated with BCMA-Directed Therapies: A Systematic Review and Meta-Analysis

**DOI:** 10.3390/cancers18040686

**Published:** 2026-02-19

**Authors:** Artur Borkowski, Ugo Giordano, Wojciech Szlasa, Krzysztof Dudek, Karolina Kędziora, Monika Mordak-Domagała, Zuzanna Dybko, Jacek Kwiatkowski, Jarosław Dybko

**Affiliations:** 1 Clinical Endocrinology Department, 4th Military Clinical Hospital, 50-981 Wrocław, Poland; 2 Department and Clinic of Endocrinology, Diabetes and Isotope Therapy, Wroclaw Medical University, 50-367 Wrocław, Poland; 3 Lower Silesia Centre for Oncology, Pulmonology and Hematology in Wrocław, 53-439 Wrocław, Poland; 4 Department of Molecular and Cellular Biology, Faculty of Pharmacy, Wroclaw Medical University, 50-556 Wrocław, Poland; 5 Centre for Research and Innovation, 4th Military Clinical Hospital, 50-981 Wrocław, Poland; 6 Faculty of Medicine, Wroclaw Medical University, 50-367 Wrocław, Poland; 7 Department of Oncology and Hematology, Faculty of Medicine, Wroclaw University of Science and Technology, 50-370 Wroclaw, Poland

**Keywords:** multiple myeloma, soluble B-cell maturation antigen, BCMA-directed therapies, prognostic biomarker

## Abstract

**Background:** B-cell maturation antigen (BCMA) directed therapies have transformed the treatment landscape for relapsed or refractory multiple myeloma (RRMM). Soluble BCMA (sBCMA), a circulating product of the membrane-bound BCMA shedding, has emerged as a potential biomarker reflecting tumor burden and disease biology. This systematic review and meta-analysis aimed to evaluate the prognostic value of baseline circulating sBCMA in patients with multiple myeloma receiving BCMA-directed therapies, with progression-free survival (PFS) as the primary endpoint. **Methods:** A systematic literature search of PubMed/MEDLINE, Embase, Scopus, and Web of Science databases was conducted in accordance with PRISMA guidelines. Studies enrolling patients with multiple myeloma treated with BCMA-directed therapies and reporting baseline circulating sBCMA measured in serum or plasma in relation to survival outcomes were included. Hazard ratios (HRs) for PFS and overall survival (OS) were pooled using random-effects models. Risk of bias was assessed using the QUIPS tool. **Results:** Four independent RRMM cohorts fulfilled the eligibility criteria and were included in the quantitative PFS meta-analysis. Elevated baseline circulating sBCMA was significantly associated with inferior PFS (pooled HR = 2.64, *p* < 0.05), with a consistent adverse prognostic direction across all studies. Moderate to substantial heterogeneity was observed (I^2^ = 63.2%), potentially reflecting differences in BCMA-directed therapy modalities across cohorts and methodological variability, including study-specific sBCMA cut-off definitions, assay procedures and sampling timepoints. Exploratory subgroup analysis suggested that the prognostic impact of baseline sBCMA on PFS may differ according to BCMA-directed therapy class. Overall risk of bias was judged as low to moderate. **Conclusions:** Elevated baseline circulating sBCMA is associated with inferior progression-free survival in patients with multiple myeloma treated with BCMA-directed therapies. These findings support the prognostic relevance of sBCMA as a risk stratification marker, although harmonization of assays and cut-offs and prospective validation are required before clinical implementation.

## Simple Summary

New treatments that target a protein called B-cell maturation antigen have greatly improved outcomes for patients with multiple myeloma, but treatment responses vary widely. Simple blood tests that could help predict treatment outcomes are still lacking. Soluble B-cell maturation antigen is a blood marker that can be measured before treatment begins and has been proposed to be associated with disease activity. In this study, we reviewed and combined available clinical evidence to evaluate whether baseline levels of this marker are linked to treatment outcomes in patients receiving B-cell maturation antigen-directed therapies. Across several independent patient groups, higher baseline levels were consistently associated with shorter periods of disease control. These results suggest that this blood marker may help identify patients at higher risk of early disease progression and could support improved risk stratification in future clinical trials and treatment planning.

## 1. Introduction

Despite rising survival rates in recent years, multiple myeloma (MM) remains an incurable plasma-cell malignancy, in which relapse and acquired drug resistance are common [1]. In response to ongoing diagnostic and therapeutic limitations, B-cell maturation antigen (BCMA) has emerged as a highly attractive target, driving major advances in the treatment of MM. The development of BCMA-directed modalities has included the introduction of chimeric antigen receptor T-cell (CAR-T) therapies, which have demonstrated marked efficacy and are increasingly being incorporated into earlier lines of treatment for relapsed or refractory multiple myeloma (RRMM) [2,3]. In parallel, bispecific antibodies (bsAbs) targeting BCMA have further expanded therapeutic options for heavily pretreated patients [4].

BCMA is a member of the tumor necrosis factor receptor superfamily and is expressed on the surface of terminally differentiated B-cells and plasma cells [5,6]. The functional protein comprises an extracellular domain of 54 amino acids, a 23-amino acid transmembrane region, and an intracellular domain of 107 amino acids [7]. Under physiological conditions, membrane-bound BCMA (mBCMA) plays a pivotal role in regulating B-cell maturation and differentiation into plasma cells. BCMA is centrally involved in plasma cell survival and clonal expansion by mediating key signaling pathways, primarily through interactions with its physiological ligands, B-cell activating factor (BAFF) and a proliferation-inducing ligand (APRIL) [8]. Functionally, BCMA acts as a non-tyrosine kinase receptor for BAFF and APRILs, which are primarily secreted in a paracrine manner by components of the bone marrow microenvironment, including stromal cells, osteoclasts, and macrophages [9,10]. Engagement of these ligands triggers receptor-mediated signaling in both normal and malignant plasma cells through key downstream pathways, notably MEK/ERK, PI3K/AKT, and NFκB cascades. Signaling through the BCMA–BAFF/APRIL axis is critical for plasma cell survival, clonal expansion, and remodeling of the tumor-supportive microenvironment, thereby contributing to multiple myeloma pathogenesis [9,11]. Receptor activity is further modulated by proteolytic processing, whereby the extracellular domain of mBCMA can be cleaved from the plasma cell surface by the ubiquitously expressed intramembranous protease γ-secretase and released into the circulation as soluble BCMA (sBCMA) [12]. In MM, sBCMA may have both immunoregulatory and therapeutic implications. By sequestering BAFF or APRIL, sBCMA can alter ligand availability, impair normal B-cell differentiation, and contribute to immunoparesis [13]. Elevated sBCMA levels may reduce the efficacy of BCMA-directed therapies by decreasing surface target density on myeloma cells and by binding therapeutic molecules in the circulation, thereby creating a functional “decoy” that can limit effective target engagement [13,14].

Soluble BCMA can be measured using several assay platforms, including ELISA-based and other immunoassay techniques. Current measurements have historically relied largely on manual ELISA methods, which are time-consuming and may be more susceptible to operator-dependent variability. Additionally, ELISA-based sBCMA measurement has been reported to show insufficient reproducibility over time in a clinical laboratory evaluation, limiting clinical implementation [15]. Recent laboratory validation of an automated microfluidics-based immunoassay (ProteinSimple ELLA) demonstrates that sBCMA can be measured on automated platforms [16]. Head-to-head comparisons of analytical performance across available assays are needed. Importantly, analytical interference can be clinically relevant. Circulating sBCMA has been reported to modulate binding of anti-BCMA agents to myeloma cells and this ligand/antigen interference may also affect binding-based assays [14]. BCMA-targeting therapeutics may spuriously lower measured sBCMA on the ProteinSimple ELLA platform, underscoring the need to consider assay design and drug/antigen interference when using sBCMA for clinical interpretation [16]. 

However, these analytical methods are not yet standardized for routine clinical use [17]. To date, no Food and Drug Administration (FDA)-approved assay is available, and harmonized cut-off values across different platforms have yet to be established, underscoring the need for assay standardization in the clinical setting.

Soluble BCMA has emerged as a promising biomarker in MM, exhibiting several favorable biological and analytical characteristics that support its potential utility in disease assessment. Serum levels of sBCMA showed no association with renal function and retained independent prognostic significance when evaluated alongside established risk factors for MM, including age, serum β2-microglobulin, hemoglobin levels, and the presence of bone disease [18]. Additionally, owing to its relatively short serum half-life (24–36 h), sBCMA has the potential to rapidly identify nonresponders and track dynamic changes in disease burden [18]. Evidence suggests that sBCMA may be particularly informative in patients with oligosecretory or non-secretory MM, as it appears to be independent of secretion status [19].

The biological basis of the adverse prognostic association with elevated sBCMA levels warrants further elucidation, but it may reflect higher tumor burden or sBCMA-mediated immune dysregulation that creates a permissive microenvironment for myeloma growth [13,20]. In addition, existing reports suggest that soluble BCMA can bind to and interfere with anti-BCMA antibodies, but the clinical impact of this interaction is not well defined and remains uncertain [14]. Collectively, these considerations provide a plausible biological rationale for the observed association between higher baseline sBCMA levels and inferior outcomes in patients receiving BCMA-directed therapies. 

This systematic review and meta-analysis aimed to evaluate the prognostic value of baseline circulating sBCMA in patients with multiple myeloma receiving BCMA-directed therapies, with progression-free survival (PFS) as the primary endpoint.

## 2. Materials and Methods

### 2.1. Protocol and Registration

This systematic review and meta-analysis was conducted in accordance with the Preferred Reporting Items for Systematic Reviews and Meta-Analyses (PRISMA) guidelines. The study was prospectively registered in the International Prospective Register of Systematic Reviews (PROSPERO) under the registration number CRD420251252066.

### 2.2. Search Strategy

A comprehensive literature search was conducted using PubMed/MEDLINE, Scopus, Embase and Web of Science databases. The search strategy incorporated terms: “multiple myeloma”, “soluble B-cell maturation antigen”, “soluble BCMA”, “sBCMA”, “serum BCMA” and “circulating BCMA”, which were combined using Boolean operators (AND, OR) and applied consistently across all databases. An example PubMed/MEDLINE search strategy was: (“Multiple Myeloma”[MeSH] OR “multiple myeloma”) AND (“soluble B-cell maturation antigen” OR “soluble BCMA” OR sBCMA OR “serum BCMA” OR “circulating BCMA” OR (soluble AND BCMA)). The query was adapted to the syntax of the other databases. The reference lists of all included studies and relevant review articles were also manually screened to identify additional eligible publications. The literature search was restricted to English-language articles published up to 15 October 2025.

### 2.3. Eligibility Criteria and Study Selection

Study screening was conducted independently by two reviewers, who assessed titles, abstracts, and subsequently full-text articles for eligibility according to predefined inclusion and exclusion criteria. Disagreements were resolved through discussion, and when consensus could not be achieved, a third reviewer acted as an adjudicator.

Inclusion criteria were defined as follows: (1) a population of adult patients with MM treated with BCMA-directed therapies, including CAR-T therapies, bispecific antibodies, or antibody–drug conjugates (ADC), either as monotherapy or in combination regimens; (2) assessment of sBCMA measured in peripheral blood (serum or plasma); (3) reporting of survival outcomes, including PFS and/or overall survival (OS), in relation to sBCMA or provision of sufficient data to allow estimation of time-to-event effect measures.

Exclusion criteria included: (1) studies involving therapies other than BCMA-directed treatments; (2) evaluation of sBCMA concentration or BCMA expression in biological material other than peripheral blood (serum or plasma); (3) lack of assessment of survival outcomes in correlation with sBCMA or absence of sufficient data to allow estimation of time-to-event effect measures; (4) duplicate publications, conference abstracts without full-text data, case reports, small case series, reviews, editorials, expert opinions, or preclinical studies.

The primary outcome of interest was progression-free survival (PFS), defined as the time from initiation of BCMA-directed therapy to documented disease progression or death from any cause, whichever occurred first. Overall survival (OS) was evaluated as a secondary, exploratory outcome.

### 2.4. Data Extraction and Risk of Bias Assessment

The following data were systematically extracted from the included studies: year of publication, study design, patient characteristics, treatment modalities including type of BCMA-directed therapy, method and timing of circulating sBCMA measurement, definition of baseline sBCMA, effect estimates, and follow-up duration. When effect estimates were not directly reported, available data were used to estimate time-to-event measures where feasible. Any discrepancies during data extraction were resolved by consensus or, if necessary, by consultation with a third reviewer. The risk of bias of the included studies was assessed using the Quality in Prognosis Studies (QUIPS) tool and was independently assessed by two reviewers, with disagreements resolved by consensus or adjudication by a third reviewer.

### 2.5. Statistical Analysis

Hazard ratios (HRs) with corresponding 95% confidence intervals (CIs) were used as the summary effect measure to evaluate the association between baseline circulating sBCMA levels and time-to-event outcomes. PFS was analyzed as the primary outcome, whereas OS was evaluated as a secondary, exploratory outcome when reported. An exploratory subgroup analysis stratified by BCMA-directed therapy class was performed post hoc to assess potential differences. When HRs were not directly reported, they were estimated from available time-to-event data using established methods.

Meta-analyses were performed using a random-effects model to account for between-study heterogeneity. Statistical heterogeneity was assessed using Cochran’s Q test and quantified with the I^2^ statistic. Potential small-study effects and publication bias were explored by visual inspection of funnel plots and Egger’s regression test, for the primary PFS analysis only. However, these assessments were interpreted with caution due to the limited number of included studies. Formal assessment of publication bias was not performed for OS because of the small number of available studies. A two-sided *p*-value < 0.05 was considered statistically significant. Pooled estimates were presented using forest plot. Sensitivity analyses were not conducted due to the limited number of eligible cohorts for HR-based PFS pooling.

## 3. Results

### 3.1. Study Selection

The database search identified a total of 893 records. After removal of duplicates, 607 records were screened based on titles and abstracts, of which 77 articles were retrieved for full-text assessment. Following full-text review, four studies met the eligibility criteria and were included in the quantitative meta-analysis of PFS [21,22,23,24]. Two of these studies additionally reported OS outcomes and were included in an exploratory OS analysis [23,24].

**Figure 1:**
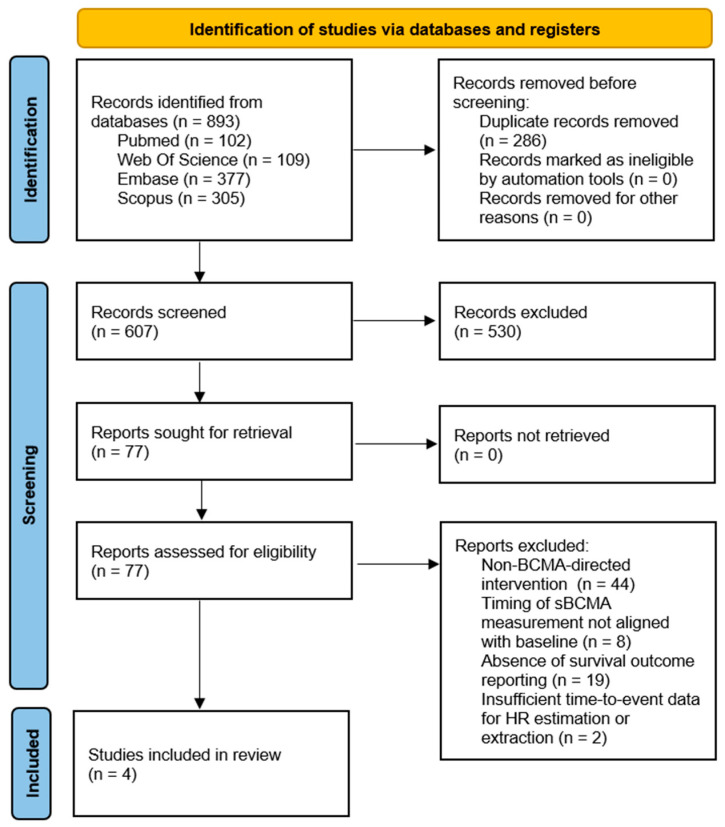
PRISMA 2020 flow diagram of study selection process.

Among the excluded full-text reports (n = 73), the most frequent reason for exclusion was the use of non-BCMA-directed interventions (n = 44; 60%), followed by lack of survival outcome reporting (n = 19; 26%), non-baseline timing of sBCMA assessment (n = 8; 11%), and insufficient time-to-event information to estimate or extract hazard ratios (n = 2; 3%). The study selection process is summarized in the PRISMA flow diagram (Figure 1).

### 3.2. Study Characteristics

The main characteristics of the four studies included in the quantitative synthesis are summarized in Table 1. The evidence base comprised four independent cohorts of patients with relapsed or refractory multiple myeloma (RRMM) treated with BCMA-directed interventions, including CAR-T products and bispecific T-cell engagers. No eligible ADC-treated cohort was available for quantitative synthesis. Lee et al. analyzed teclistamab-treated patients from the MajesTEC-1 phase 1/2 clinical trial dataset, whereas Freeman et al. and Wiemers et al. evaluated real-world cohorts receiving standard-of-care CAR-T products [21,23,24]. Lon et al. contributed data from a pooled population exposure–response analysis of elranatamab across MagnetisMM-1, -2, -3, and -9 clinical trials [22]. Across all studies, baseline circulating sBCMA was measured in serum or plasma from peripheral blood prior to initiation of BCMA-directed therapy. The detection rate of circulating sBCMA was not consistently reported across the included studies. Lon et al. noted data points with sBCMA values below the assay lower limit of quantification (0.08 ng/mL), which were imputed as 0.001 ng/mL for plotting purposes, without reporting the frequency or reasons for these below-quantification measurements [22]. Prior exposure to BCMA-directed therapy varied across cohorts. It was not permitted in MajesTEC-1 trial, while it was present in a subset of patients in the Freeman et al. and Lon et al. cohorts (13% and 31%, respectively) [22,24,25]. This variable was not explicitly reported in Wiemers et al. [23]. Prognostic stratification was based on predefined or median-derived sBCMA cut-offs, depending on study design. Although follow-up duration varied across cohorts, all were sufficient to support HR-based time-to-event analyses for survival outcomes. 

### 3.3. Meta-Analysis of Progression-Free Survival

All four meta-analysis cohorts reported hazard ratios for PFS or provided sufficient data for HR estimation. Pooled analysis using a random-effects model yielded an overall hazard ratio of 2.64 (95% CI not crossing the null; *p* < 0.05), indicating a statistically significant adverse prognostic association between baseline circulating sBCMA and progression-free survival in patients receiving BCMA-directed treatment. This direction of effect was consistent across the included studies, each showing a trend toward shorter PFS in patients with higher baseline circulating sBCMA. Collectively, these findings support the prognostic relevance of baseline sBCMA in time-to-event analyses for patients receiving BCMA-directed therapy.

**Figure 2:**
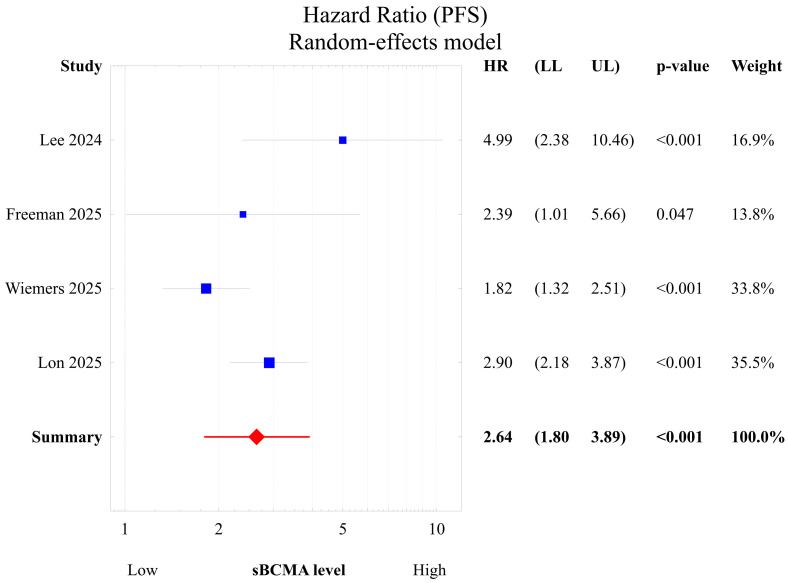
Forest plot showing hazard ratios for progression-free survival (PFS) comparing patients with high versus low baseline circulating sBCMA levels across individual studies (Lee et al. [21], Lon et al. [22], Wiemers et al. [23], Freeman et al. [24]) and the pooled random-effects estimate. Elevated baseline sBCMA was consistently associated with an inferior PFS. Blue squares denote individual study estimates (size proportional to weight) and the red diamond represents the pooled random-effects estimate.

Statistical heterogeneity among the included studies was moderate to substantial (I^2^ = 63.2%, Cochran’s Q = 8.14, df = 3, *p* = 0.043). Pooled effect estimates were therefore derived using a random-effects model, accounting for between-study variability. The forest plot illustrating individual and pooled effect estimates is shown in Figure 2.

#### Exploratory Subgroup Analysis by Therapy Modality

An exploratory subgroup analysis was performed to compare the prognostic association between baseline circulating sBCMA and progression-free survival according to BCMA-directed therapy modality (CAR-T vs bsAbs). In patients treated with bsAbs, elevated baseline sBCMA was associated with a stronger adverse prognostic effect on PFS (pooled HR = 3.40), whereas a more modest association was observed in CAR-T-treated cohorts (pooled HR = 1.88). The between-group heterogeneity test indicated a statistically significant difference in effect size between therapy modalities (Q = 4.14, df = 1, *p* = 0.042). Within-group heterogeneity was low and not statistically significant for either subgroup (Q for bsAbs: *p* = 0.32; Q for CAR-T: *p* = 0.56). The pooled subgroup-specific hazard ratios and heterogeneity estimates are summarized in Table 2, while the corresponding forest plot is provided in Supplementary Figure S1. Given the limited number of studies contributing to each subgroup, these findings should be interpreted as exploratory and hypothesis-generating.

### 3.4. Exploratory Analysis of Overall Survival

Overall survival was reported in two studies and was therefore analyzed as a secondary, exploratory outcome. Pooled analysis demonstrated an association between higher baseline circulating sBCMA levels and inferior overall survival (HR = 1.28, 95% CI 1.03–1.59; *p* = 0.027). Given the limited number of available studies, no funnel plot or formal publication-bias testing was performed for OS; therefore, this observation should be interpreted cautiously.

### 3.5. Risk of Bias Assessment

The risk of bias of the included studies was assessed using the Quality in Prognosis Studies (QUIPS) tool. Overall, the included studies were judged to have low to moderate risk of bias across most QUIPS domains. The main sources of potential bias were related to variability in the definition of baseline sBCMA cut-off values. No high risk of bias was identified in the domains of study participation or outcome measurement, and no study was excluded on the basis of methodological quality.

Potential publication bias for the primary PFS analysis was explored using funnel plot inspection and Egger’s regression test, which did not indicate marked asymmetry. However, these findings should be interpreted with caution due to the limited number of included studies. Formal assessment of publication bias was not performed for OS because only two studies were available.

A structured summary of QUIPS domain-level judgments is provided in Supplementary Table S1, and the funnel plot for the PFS analysis is presented as Supplementary Figure S2. 

## 4. Discussion

In the context of the expanding use of BCMA-directed therapies and the need for reliable treatment-specific prognostic markers, the present meta-analysis evaluated the prognostic value of baseline circulating sBCMA. Pooled evidence from four independent RRMM cohorts indicates that elevated baseline circulating sBCMA is associated with significantly inferior PFS in patients receiving BCMA-directed therapies (HR = 2.64). The adverse prognostic direction was consistent across all included studies, strengthening the credibility of this signal. Prognostic stratification relied on study-level sBCMA cut-offs rather than a harmonized threshold, therefore inter-cohort differences likely contributed to heterogeneity, but they do not negate the overall interpretation that sBCMA captures biologically meaningful risk variance relevant to BCMA-targeted treatment outcomes. Exploratory subgroup analysis further suggested that the magnitude of the prognostic association between baseline sBCMA and PFS may differ according to the class of BCMA-directed therapy, appearing stronger in cohorts treated with bispecific antibodies than with CAR-T cell therapies. An exploratory pooling of overall survival from two studies also suggested inferior OS in patients with higher baseline sBCMA (HR = 1.28, *p* = 0.027). However, this estimate remains hypothesis-generating due to the limited number of OS-reporting cohorts and should be interpreted cautiously. Notably, the evidence base included both clinical trial populations and real-world cohorts, and sBCMA was consistently assessed before initiation of BCMA-directed therapy in heavily pretreated RRMM despite modest between-study differences in sampling timepoints, prior therapy exposure, and follow-up. In the absence of patient-level data and harmonized study-level cut-offs, sBCMA should currently be interpreted as a relative risk stratification marker rather than a fixed clinical decision threshold

The observed heterogeneity (I^2^ = 63.2%) likely reflects both clinical and methodological variability across cohorts. Contributing factors may include differences in BCMA-directed therapy modalities (CAR-T versus bispecific antibodies), baseline patient risk profiles (e.g., prior treatment exposure and trial-derived versus real-world populations), and non-uniform sBCMA assessment approaches (study-specific cut-offs, assay procedures, and sampling timepoints). Within this framework, differences between the two real-world CAR-T cohorts provide a concrete example of clinically driven heterogeneity. In the Freeman et al. cohort, ide-cel was the predominant product (68% of treated patients), whereas cilta-cel accounted for 32%, and survival follow-up was relatively mature, with a median follow-up of approximately 15.5 months [24]. In contrast, the Wiemers et al. cohort enrolled a more balanced distribution of CAR-T products (ide-cel n = 35 vs cilta-cel n = 38) but with shorter follow-up (median follow-up for PFS of 6.1 months; for OS of 8.2 months) [23]. While both studies demonstrated a consistent adverse prognostic direction of high baseline circulating sBCMA, the magnitude of PFS separation between low vs high sBCMA appeared numerically larger among cilta-cel recipients in the Freeman et al. dataset, where 12-month PFS estimates were reported as 85% vs 30% (low vs high sBCMA), compared with 58% vs 38% in the ide-cel subgroup. Nevertheless, this difference did not reach statistical significance. Regarding overall survival in Freeman et al., high baseline sBCMA did not significantly stratify OS in the overall cohort or among ide-cel recipients, while a visible, directional OS separation was observed in patients treated with cilta-cel [24].

Two additional studies reported associations between baseline circulating sBCMA levels and survival outcomes: Caillot et al., evaluating patients treated with idecabtagene vicleucel in a small patient cohort, and Oliver-Caldés et al., analyzing an academic BCMA-directed CAR-T product (ARI0002h—cesnicabtagene autoleucel) [26,27]. However, the absence of hazard ratio estimates or sufficient time-to-event data precluded quantitative pooling. Caillot et al. reported that patients with higher baseline sBCMA tended to experience earlier relapse and shorter progression-free intervals, consistent with a more aggressive clinical trajectory in heavily pretreated RRMM [26]. Oliver-Caldés et al. similarly observed that patients with elevated baseline sBCMA demonstrated earlier events across DOR, PFS, and OS endpoints, reinforcing that sBCMA reflects meaningful between-patient risk variance [27]. Importantly, the survival trends described in both studies align with the adverse prognostic direction identified in the present PFS meta-analysis, supporting a concordant overall signal despite methodological differences and limited cohort sizes.

The biological plausibility of this adverse prognostic signal is supported by prior evidence linking circulating sBCMA to disease burden and BCMA-axis biology. Across several studies, serum sBCMA concentrations were consistently higher in patients with multiple myeloma than in healthy donors [18,20,28]. Importantly, higher circulating sBCMA levels were observed in patients with greater disease burden, reflecting a close relationship with myeloma cell load and the proportion of bone marrow plasma cells [13,18,20]. Mechanistically, BCMA shedding and soluble ligand binding provide additional context for how elevated baseline sBCMA could reflect both tumor biology and treatment-relevant target dynamics. BCMA signaling intersects with several pathways central to multiple myeloma biology, particularly the NF-κB axis, which regulates genes involved in plasma cell survival, proliferation, and disease progression [29].Proteolytic shedding of membrane-bound BCMA reduces receptor density on myeloma cells, thereby attenuating BCMA-dependent signaling while simultaneously increasing circulating soluble BCMA [13]. This process has important immunoregulatory and therapeutic implications. Soluble BCMA can bind and sequester BAFF and APRIL, thereby altering ligand availability within the bone marrow microenvironment. By limiting APRIL signaling and redistributing BAFF, elevated sBCMA may perturb normal humoral homeostasis, what have been associated with suppression of uninvolved immunoglobulin production, including IgA deficiency. Moreover, increased circulating sBCMA released by myeloma cells may reduce the physiological BAFF pool available to normal B-cells, impairing their maturation into antibody-secreting plasma cells and contributing to immunoparesis in multiple myeloma [28,30,31]. From a therapeutic perspective, increased BCMA shedding may reduce the efficacy of BCMA-directed agents by lowering surface target density and by sequestering therapeutic antibodies or bispecific constructs in the circulation [13]. Some studies described interference of elevated sBCMA with antibody binding, supporting the plausibility of a clinically relevant “sink” effect under high-exposure conditions [14,21]. In contrast, one study reported preserved anti-myeloma CAR-T activity in the presence of sBCMA [32]. However, this analysis was performed using samples with relatively low sBCMA concentrations, which may limit generalizability to the broader RRMM population. Consequently, strategies aimed at modulating BCMA shedding, such as γ-secretase inhibitors, have been proposed to enhance surface BCMA expression and reduce circulating “decoy” activity, with the goal of improving the efficacy of BCMA-targeted modalities, and are under clinical investigation [33,34].

An important unresolved question is whether the association between higher baseline sBCMA and inferior PFS is driven by differences in treatment response dynamics. Response endpoints were reported inconsistently across the included studies, precluding a quantitative synthesis. Descriptively, the pooled elranatamab analysis by Lon et al. found that lower baseline sBCMA was associated with longer DOR (*p* = 0.012), and Oliver-Caldés et al. similarly reported that higher baseline sBCMA correlated with a shorter time to event in DOR (*p* < 0.001) [22,27]. Lee et al. reported lower baseline sBCMA levels among teclistamab responders than non-responders, suggesting an association between baseline sBCMA and ORR [21]. Addressing this question will require harmonized study designs and standardized reporting of response outcomes alongside baseline sBCMA.

Current MM staging systems were developed using datasets collected before the introduction and broad clinical adoption of BCMA-directed therapies, meaning they reflect historical rather than contemporary BCMA-directed treatment risk trajectories [35]. This underscores a critical need to evaluate circulating sBCMA specifically in the context of modern BCMA-targeted treatment pathways, where baseline risk may interact with therapy efficacy differently than in legacy regimens. Guo et al. reported a correlation between baseline circulating sBCMA and advanced disease risk categories, supporting its role as an independent prognostic marker in MM [20]. However, because the study population combined newly diagnosed and RRMM populations with heterogeneous treatment exposures, it was not methodologically compatible with our quantitative synthesis. Nevertheless, the direction of the reported prognostic signal was aligned with our findings, offering supportive, but non-pooled context.

More broadly, the present results should be interpreted alongside contemporary biomarker development efforts in MM, where transcriptomic and genomic readouts are increasingly used but may not fully capture protein-level target dynamics relevant to BCMA-directed therapies. In MM, biomarker development has increasingly shifted toward genomic and transcriptomic readouts—ranging from standard cytogenetics and gene-expression signatures to high-resolution RNA-sequencing and, more recently, single-cell RNA-sequencing (scRNA-seq) approaches that aim to resolve clonal architecture and microenvironmental states that may be obscured in bulk analyses [36]. Within the BCMA axis, transcript-level variability has also been linked to genomic events. Lee et al. linked genomic structural variation involving TNFRSF17, the gene encoding BCMA, and the transcriptional regulator POU2AF1 to higher BCMA transcript expression [21]. However, transcript abundance does not necessarily translate into functional target availability or therapeutic vulnerability, because clinically meaningful variation can also arise through post-synthesis protein processing. A BCMA-specific example is gamma-secretase-mediated cleavage of membrane BCMA, which generates circulating sBCMA. Accordingly, in the MajesTEC-1 cohort analyzed in the same study, sBCMA showed limited concordance with membrane BCMA and bone marrow plasma cell burden, underscoring that protein-level processes such as shedding can decouple soluble biomarkers from transcript abundance or surface expression alone [21].

This systematic review and meta-analysis demonstrates several key strengths, including a focused evaluation of baseline circulating sBCMA measured prior to BCMA-directed therapy, establishing a consistent temporal reference point for prognostic interpretation. To our knowledge, based on comprehensive literature searches, the present study represents the first systematic review and meta-analysis specifically evaluating circulating sBCMA as a prognostic biomarker for survival outcomes in multiple myeloma. Clinical and analytical specificity was maintained by restricting inclusion to serum or plasma sBCMA assessments, while avoiding the methodological mixing of BCMA signals derived solely from bone marrow or other tissue-restricted matrices. The adverse prognostic direction associated with elevated baseline sBCMA was preserved across independent trial-derived datasets and real-world populations, reinforcing the stability of the prognostic signal across mechanistically distinct BCMA-directed modalities. Importantly, although the individual cohorts consistently reported an adverse prognostic association, sBCMA was not the primary focus in several of the included studies. Thus, this meta-analysis adds value by consolidating dispersed evidence and providing a dedicated synthesis of the prognostic value of baseline sBCMA. The present meta-analysis has several limitations related to the available evidence base. A major limitation is the small number of independent cohorts available for quantitative synthesis (n = 4). While the pooled association between elevated baseline sBCMA and inferior PFS was statistically significant, meta-analyses based on a small number of studies provide less precise effect-size estimates and have limited statistical power for detecting between-study differences. This constraint particularly affects the robustness of exploratory subgroup comparisons and the interpretability of formal publication bias assessments. Further confirmation in larger, independent studies is warranted. In addition, no eligible antibody–drug conjugate cohort was identified for quantitative synthesis, limiting the applicability of these findings beyond CAR-T and BsAbs settings. Second, survival analyses relied on study-level sBCMA cut-offs rather than individual patient-level data, precluding verification of proportional-hazards assumptions and uniform multivariable adjustment. Finally, methodological differences in assay procedures and cut-off definitions may underlie part of the observed between-study heterogeneity.

Future research efforts should prioritize validation in prospective BCMA-treated cohorts, harmonization of sBCMA cut-offs using transparent and reproducible derivation strategies, and exploration of next-generation risk models conceptualized in the era of BCMA-targeted treatment. Particular attention should be directed toward the integration of sBCMA into multivariable risk frameworks that combine tumor biology, treatment exposure, and established prognostic variables. These advances would help define the clinical utility of circulating sBCMA for therapeutic risk stratification and enable optimized, biomarker-informed patient grouping in contemporary MM care.

## 5. Conclusions

Collectively, the present systematic review and meta-analysis supports the prognostic relevance of elevated baseline circulating sBCMA for inferior PFS in multiple myeloma patients receiving BCMA-directed therapies. Exploratory observations suggest potential variability by therapy class and a possible association with overall survival. However, findings beyond the primary PFS endpoint should be considered hypothesis-generating given the limited number of available cohorts and study-specific cut-offs. Prospective validation and harmonized cut-off derivation are warranted to define the clinical utility of sBCMA for contemporary, BCMA-era risk stratification.

## Figures and Tables

**Table 1:** Characteristics of studies included in the quantitative synthesis. * Data derived from MajesTEC-1 study. ** Lon et al. did not report the median number of prior therapy lines, however 51% of the study population had received ≥5 prior lines of treatment. *** In Lon et al., follow-up was determined by the data cut-off time points of the contributing clinical trials within the MagnetisMM program. Median follow-up value was not reported.

Study (Year)	Study Design	Multiple Myeloma Condition	Number of Patients (n)	Prior Therapy Lines—Median, (range)	Prior Exposure to BCMA-Directed Therapy n (%)	BCMA-Directed Therapy Type	Specific Therapeutic Agent	sBCMA Cut-Off Strategy	sBCMA Cut-Off Value (ng/mL)	Reported Outcomes (PFS, OS)	Median Follow-Up (Months), Range	Timing of sBCMA Measurement
Lee et al. [21] (2024)	Post hoc analysis (clinical trial dataset)	RRMM	163	5 (2–14) *	0 *	bsAbs	Teclistamab	Predefined	400	PFS	14.1 (0.3–24.4) *	Trial baseline (protocol-defined, pre-first dose)
Lon et al. [22] (2024)	Exposure–response analysis (pooled clinical trials dataset)	RRMM	312	NR (51%—n ≥ 5) **	31	bsAbs	Elranatamab	Predefined	100	PFS	NR ***	Trial baseline (protocol-defined, pre-first dose)
Wiemers et al. [23] (2025)	Observational cohort (single-center)	RRMM	73	7 (1–17)	NR	CAR-T	Ide-cel; cilta-cel	Predefined	175	PFS, OS	6.1 (for PFS), 8.2 (for OS)	at leukapheresis
Freeman et al. [24] (2025)	Observational cohort (multicenter)	RRMM	183	6 (4–13)	13	CAR-T	Ide-cel; cilta-cel	Median-derived	97.1	PFS, OS	15.5 (1.07–35.91)	Pre-LD

Abbreviations: RRMM, relapsed/refractory multiple myeloma; bsAbs, bispecific antibodies; CAR-T, chimeric antigen receptor T-cell therapy; sBCMA, soluble B-cell maturation antigen; PFS, progression-free survival; OS, overall survival; Ide-cel, Idecabtagene vicleucel; cilta-cel, Ciltacabtagene autoleucel, LD, lymphodepletion; NR, not reported.

**Table 2:** Exploratory subgroup analysis of the association between baseline sBCMA and progression-free survival according to BCMA-directed therapy class. Heterogeneity was assessed using Cochran’s Q test. Hazard ratios were pooled using a random-effects model. The “Overall” row reflects the subgroup model combining therapy classes. Accordingly, the corresponding HR may differ from the overall pooled HR obtained from a single random-effects model that combines all four cohorts without subgrouping.

Subgroup by Therapy Class	Number of Studies (n)	Pooled HR (95% CI)	*p*-Value	Within-Subgroup Heterogeneity (Q, df, *p*)	Between-Subgroup Heterogeneity (Q, df, *p*)
bsAbs	2	3.40 (2.10–5.52)	<0.001	Q = 1.000, df = 1, *p* = 0.317	NA
CAR-T	2	1.88 (1.39–2.54)	<0.001	Q = 0.337, df = 1, *p* = 0.561	NA
Overall	4	2.22 (1.72–2.87)	<0.001	Q = 1.34; df = 2, *p* = 0.51	Q = 4.14, df = 1, *p* = 0.042

Abbreviations: HR, hazard ratio; CI, confidence interval; sBCMA, soluble B-cell maturation antigen; bsAbs, bispecific antibodies; CAR-T, chimeric antigen receptor T-cell therapy; NA, not applicable.

## Data Availability

The data supporting the findings of this study are available from the corresponding author upon reasonable request.

## Supplementary Materials

The following supporting information can be downloaded at: https://www.mdpi.com/article/10.3390/cancers18040686/s1, Figure S1: Exploratory subgroup analysis of progression-free survival (PFS) by BCMA-directed therapy modality. Forest plot showing hazard ratios (HRs) for PFS associated with baseline sBCMA levels in CAR-T-treated and bispecific antibody-treated cohorts; Figure S2: Funnel plot for the primary PFS meta-analysis showing no marked asymmetry. Interpretation is limited by the small number of included studies (n = 4); Table S1: Risk of bias assessment of the included studies using the Quality In Prognosis Studies (QUIPS) tool. Six domains were evaluated: study participation, study attrition, prognostic factor measurement, outcome measurement, study confounding, and statistical analysis and reporting. Each domain was rated as low, moderate, or high risk of bias. PFS, progression-free survival; OS, overall survival; sBCMA, soluble B-cell maturation antigen; Table S2: PRISMA 2020 checklist for the present systematic review and meta-analysis [37].

## Abbreviations

The following abbreviations are used in this manuscript:

BCMA	B-cell maturation antigen
RRMM	Relapsed/refractory multiple myeloma
sBCMA	Soluble B-cell maturation antigen
HR	Hazard ratio
CI	Confidence interval
PFS	Progression-free survival
OS	Overall survival
QUIPS	Quality in Prognosis Studies
MM	Multiple myeloma
CAR-T	Chimeric antigen receptor T-cell
bsAbs	Bispecific antibodies
mBCMA	Membrane-bound B-cell maturation antigen
