# Supplementary material for: Soluble B-Cell Maturation Antigen as a Prognostic Marker for Progression-Free Survival in Multiple Myeloma Treated with BCMA-Directed Therapies: A Systematic Review and Meta-Analysis
Source: Cancers (Basel). 2026 Feb 19;18(4):686. doi: 10.3390/cancers18040686 (PMC12938959; doi:10.3390/cancers18040686)
Supplement: Supplementary file 1 [file cancers-18-00686-s001.zip › supplementary files - captions.pdf]

Supplementary Figure S1. Exploratory subgroup analysis of progression-free survival (PFS) by BCMA-directed therapy modality. Forest plot showing hazard ratios (HRs) for PFS associated with baseline sBCMA levels in CAR-T–treated and bispecific antibody–treated cohorts.

Supplementary Figure S2. Funnel plot for the primary PFS meta-analysis showing no marked asymmetry. Interpretation is limited by the small number of included studies (n = 4).

Supplementary Table S1. Risk of bias assessment of the included studies using the Quality In Prognosis Studies (QUIPS) tool. Six domains were evaluated: study participation, study attrition, prognostic factor measurement, outcome measurement, study confounding, and statistical analysis and reporting. Each domain was rated as low, moderate, or high risk of bias. PFS = progression-free survival; OS = overall survival; sBCMA = soluble B-cell maturation antigen.

Supplementary Table S2. PRISMA 2020 checklist for the present systematic review and meta-analysis.
