# Supplementary material for: Soluble B-Cell Maturation Antigen as a Prognostic Marker for Progression-Free Survival in Multiple Myeloma Treated with BCMA-Directed Therapies: A Systematic Review and Meta-Analysis
Source: Cancers (Basel). 2026 Feb 19;18(4):686. doi: 10.3390/cancers18040686 (PMC12938959; doi:10.3390/cancers18040686)
Supplement: Supplementary file 1 [file cancers-18-00686-s001.zip › TableS1.pdf]

Study	Study Participation	Study Attrition	Prognostic Factor Measurement (sBCMA)	Outcome Measurement (PFS)	Study Confounding	Statistical Analysis and Reporting	Overall Risk of Bias
Wiemers et al.	Low	Low	Moderate	Low	Moderate	Low	Low–Moderate
Freeman et al.	Low	Low	Moderate	Low	Moderate	Low	Low–Moderate
Lee et al.	Low	Moderate	Moderate	Low	Moderate	Moderate	Moderate
Lon et al.	Low	Low	Moderate	Low	Moderate	Low	Low–Moderate

Supplementary Table S1. Risk of bias assessment of the included studies using the Quality In Prognosis Studies (QUIPS) tool. Six domains were evaluated: study participation, study attrition, prognostic factor measurement, outcome measurement, study confounding, and statistical analysis and reporting. Each domain was rated as low, moderate, or high risk of bias. PFS, progression-free survival; OS, overall survival; sBCMA, soluble B-cell maturation antigen.
