# Supplementary figures and images for: Soluble B-Cell Maturation Antigen as a Prognostic Marker for Progression-Free Survival in Multiple Myeloma Treated with BCMA-Directed Therapies: A Systematic Review and Meta-Analysis
Source: Cancers (Basel). 2026 Feb 19;18(4):686. doi: 10.3390/cancers18040686 (PMC12938959; doi:10.3390/cancers18040686)

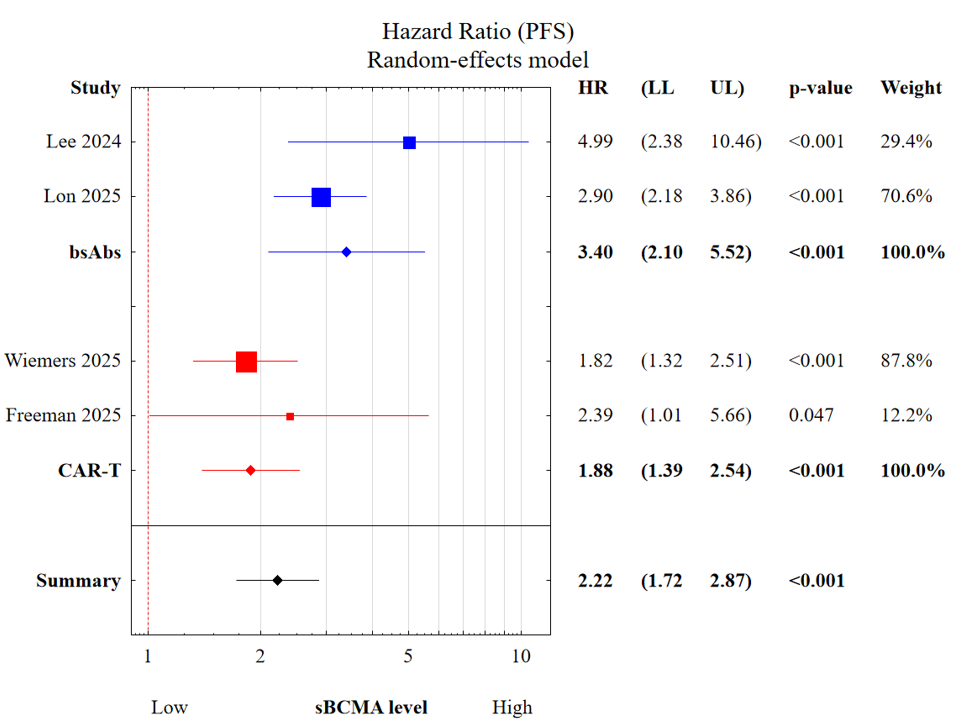

Supplement: Supplementary file 1 [file cancers-18-00686-s001.zip › FigureS1.png]

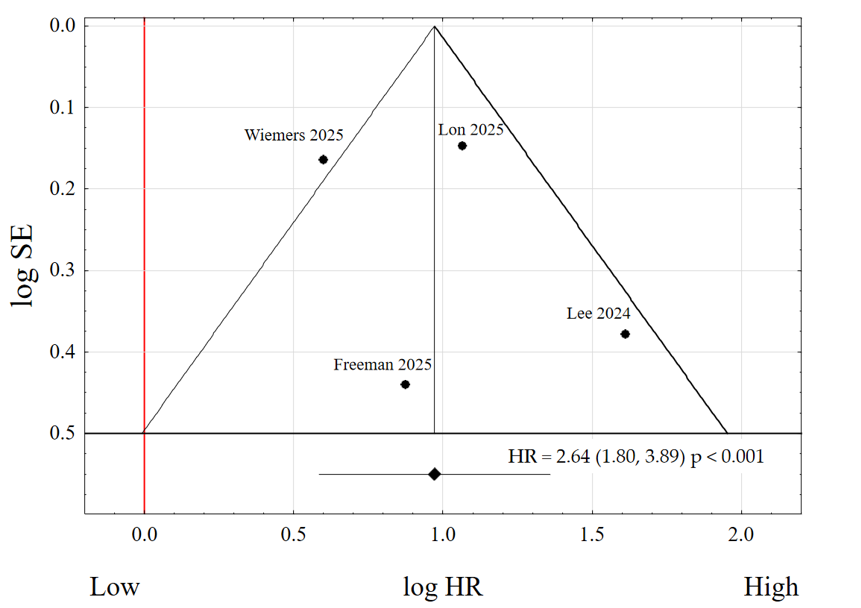

Supplement: Supplementary file 1 [file cancers-18-00686-s001.zip › FigureS2.png]
